# Exosome‐Guided Phenotypic Switch of M1 to M2 Macrophages for Cutaneous Wound Healing

**DOI:** 10.1002/advs.201900513

**Published:** 2019-08-27

**Authors:** Hyosuk Kim, Sun Young Wang, Gijung Kwak, Yoosoo Yang, Ick Chan Kwon, Sun Hwa Kim

**Affiliations:** ^1^ KU‐KIST Graduate School of Converging Science and Technology Korea University Seoul 02841 Republic of Korea; ^2^ Center for Theragnosis Biomedical Research Institute Korea Institute of Science and Technology (KIST) Seoul 02792 Republic of Korea

**Keywords:** cutaneous wound healing, direct cell reprogramming, exosomes, macrophage phenotype switch

## Abstract

Macrophages (Mϕs) critically contribute to wound healing by coordinating inflammatory, proliferative, and angiogenic processes. A proper switch from proinflammatory M1 to anti‐inflammatory M2 dominant Mϕs accelerates the wound healing processes leading to favorable wound‐care outcomes. Herein, an exosome‐guided cell reprogramming technique is proposed to directly convert M1 to M2 Mϕs for effective wound management. The M2 Mϕ‐derived exosomes (M2‐Exo) induce a complete conversion of M1 to M2 Mϕs in vitro. The reprogrammed M2 Mϕs turn Arginase (M2‐marker) and iNOS (M1‐marker) on and off, respectively, and exhibit distinct phenotypic and functional features of M2 Mϕs. M2‐Exo has not only Mϕ reprogramming factors but also various cytokines and growth factors promoting wound repair. After subcutaneous administration of M2‐Exo into the wound edge, the local populations of M1 and M2 Mϕs are markedly decreased and increased, respectively, showing a successful exosome‐guided switch to M2 Mϕ polarization. The direct conversion of M1 to M2 Mϕs at the wound site accelerates wound healing by enhancing angiogenesis, re‐epithelialization, and collagen deposition. The Mϕ phenotype switching induced by exosomes possessing the excellent cell reprogramming capability and innate biocompatibility can be a promising therapeutic approach for various inflammation‐associated disorders by regulating the balance between pro‐ versus anti‐inflammatory Mϕs.

## Graphical abstract

**An exosome‐guided cell reprogramming technique to directly convert M1 to M2 macrophages in situ** is introduced as a promising therapeutic strategy for effective and rapid wound healing. With excellent cell reprogramming capabilities and biocompatibility, the exosome‐guided macrophage reprogramming technique can be applied not only to wound healing but also to various inflammation related diseases.

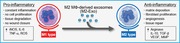

## 1 Introduction

Wound healing is a highly programmed process involving a well‐orchestrated series of cellular and molecular events: hemostasis, inflammation, proliferation, and remodeling phases. In particular, the transition from an initial inflammatory phase to a proliferative phase is a critical regulatory point that determines wound healing outcomes.^1^ Among immune cells in the wound, macrophages (Mφs) are known as key players facilitating the inflammatory‐proliferation phase transition. Mφs which are activated by specific environmental signals are broadly categorized into two main subtypes: classically activated M1 Mφ with proinflammatory properties and alternatively activated M2 Mφ exhibiting anti‐inflammatory and prowound healing functions.^2^ Thus, controlling Mφ phenotypic switch from M1 to M2 at specific time points offers a promising solution for the transition from the inflammation to the proliferation phase of wound repair. However, many attempts focus on strategies associated with the augmentation of M2 Mφ activity, the recruitment of more M2 Mφs, or the addition of exogenous M2 Mφs, while remaining unaffected in M1 Mφs.^3^ When proinflammatory M1 Mφs persist without transitioning to anti‐inflammatory phenotypes, which is believed to contribute to impaired wound healing.

**Figure 1:**
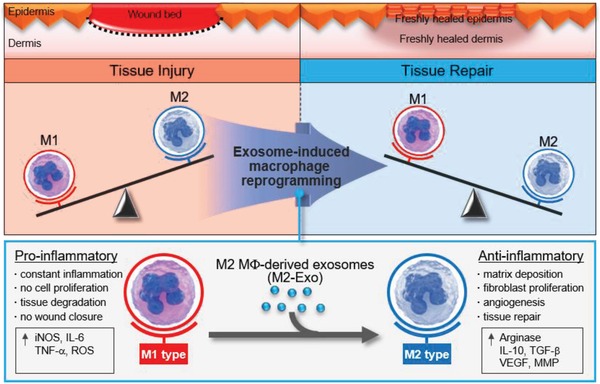
Schematic illustration of exosome‐guided macrophage reprogramming. M2 Mϕ‐derived exosomes (M2‐Exo) can induce a conversion of M1 to M2 Mϕs and accelerate cutaneous wound healing.

In order to reinforce the M2 Mφ phenotype on‐site together with attenuating M1 Mφs, in this study we propose exosome‐guided phenotypic switching as a method to directly reprogram resident M1 into M2 Mφs (**Figure** **1**). Exosomes, cell‐derived extracellular nanovesicles with a diameter 30–150 nm, are known to mediate cell‐to‐cell communication by transferring a particular composition of proteins, lipids, RNAs, and DNAs to the recipient cells.^4^ In this regard, the exosome‐mediated cell–cell communication is described as being a universal way to interact between neighboring cells and influence the behavior of recipient cells. In the field of cell‐based regenerative medicine, exosomes are recently explored as a potential tool for cell‐free therapy, taking and delivering specific cellular signals, rather than the cells.^5^ Herein, we found that the exosomes isolated from alternatively activated M2 Mφs (M2‐Exo) could induce direct reprogramming of M1 to M2 Mφs achieving extremely high conversion efficiency (≈100%). The reprogrammed M2 Mφs (RM2) recovered the capability to produce matrix metalloproteinase (MMP) and vascular endothelial growth factor (VEGF) proteins similar to alternatively activated M2 Mφs, essential in the proliferative phases characterized by angiogenesis and re‐epithelialization. M1‐Exo and M2 Exo were produced respectively by the classically activated M1 and the alternatively activated M2 state generated from mouse bone marrow‐derived hematopoietic precursors. After characterizing their biophysical and biochemical properties, exosomes were applied to cutaneous wounds on mice to drive in situ exosome‐guided Mφ phenotypic switch and to further exploit its physiological therapeutic actions in wound healing.

## 2 Results and Discussion

### 2.1 Establishment of Mouse M1 and M2 Mϕs and Characterization of Exosomes Derived from Mϕs

**Figure 2:**
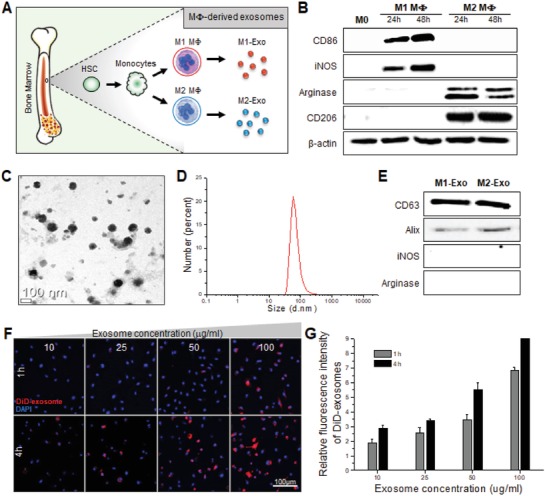
Establishment of M1 and M2 Mϕs and characterization of M2‐Exo. A) Schematic diagram of Mϕs and exosomes preparation. B) Western blot analysis demonstrating differences in expression of Mϕ markers by polarization time. C) Representative TEM image of exosomes after negatively staining with uranium acetate. D) Size distribution diagram of M2‐Exo measured using dynamic light scattering. E) Western blot analysis of exosomes. Equal amounts of total proteins extracted from exosomes were immunoblotted for CD63, Alix, iNOS, and Arginase. F) Confocal images of M1 Mϕs after 1 or 4 h incubation with 10, 25, 50, and 100 µg mL^−1^ of DiD‐labeled M2‐Exo, respectively. Images of DiD‐labeled M2‐Exo (red) with DAPI (blue) were visualized by merging the confocal images. G) Relative fluorescence intensity of DiD‐labeled M2‐Exo internalized in M1 Mϕs.

Prior to the study on exosome‐guided direct cell conversion, exosomes were prepared from both classically activated M1 and alternatively activated M2 Mφs. There have been several reports that the polarization and metabolism of Mφs are somewhat different depending on the mouse strain.^6^ Thus, herein all macrophages derived from primary monocytes were consistently prepared from BALB/c mice. In order to obtain Mφ‐derived exosomes, primary monocytes were differentiated from bone marrow‐derived hematopoietic stem cells (HSCs) and then activated to acquire different functional phenotypes of Mφs (**Figure** **2**A). First, bone marrow‐derived hematopoietic precursors were isolated from mouse femur and tibia and incubated in the presence of monocyte‐colony stimulating factor (M‐CSF) for 7 d to facilitate monocyte differentiation. The primary cultured monocytes with a round and flattened morphology began to adhere to the petri dish and were further differentiated into uncommitted Mφs (M0). Fluorescence‐activated cell sorting (FACS) analysis showed that over 95% of HSCs were differentiated into M0 Mφs expressing F4/80, a specific marker of Mφs (Data not shown). Mφs are capable of adopting distinct phenotypes and functions that are shaped by their surrounding environment of the organ of residence. It is known that M1 phenotype is induced by toll‐like receptor agonists or Th1 cytokines such as interferon‐gamma (IFN‐γ) and M2 phenotype is activated by Th2 cytokines such as interleukin 10 (IL10) and IL4.^7^, ^8^ For in vitro Mφ activation to acquire proinflammatory M1 and anti‐inflammatory M2 phenotype, the uncommitted M0 Mφs were stimulated by IFN‐γ and IL4, respectively. Morphological differences were observed in the activated Mφs between different phenotypes, which were consistent with previous observations;^9^ M1 phenotype displayed the characteristic fried egg shape morphology, while M2 phenotype showed a mixed population of fried egg‐ and spindle‐shaped cells (Figure S1, Supporting Information). Western blot analysis showed that the representative M1 (CD86 and iNOS) and M2 (Arginase and CD206) markers were clearly identified after 1 d exposure to cytokines in the activated Mφs of different phenotypes, respectively (Figure 2B).

After isolation of exosomes from the supernatant of the activated Mφs, the purified Mφ‐derived exosomes were characterized in terms of morphology, size, and surface marker expression. The transmission electron microscopy (TEM) observation revealed that the Mφ‐derived exosomes had a round vesicle‐shaped morphology with a mean diameter of 69.74 ± 17.46 nm (Figure 2C,D). Both M1‐Exo and M2‐Exo were positive for exosomal marker proteins CD63 and Alix (Figure 2E). These results supported that Mφ‐derived exosomes were successfully prepared with typical morphological and molecular features of exosomes. Since exosomes are known to participate in the exchange of some phenotypic traits between cells,^10^ the exosomal levels of iNOS or Arginase were also analyzed to exclude the possibility of a simple transfer of these Mφ‐specific marker proteins from donor to recipient cells via exosomes. Notably, none of the Mφ‐derived exosomes contained classic iNOS and Arginase phenotypic markers of activated Mφs. In addition, successful delivery of exosomes into target cells of interest is a prerequisite to perform an exosome‐guided direct cell reprogramming. However, it is known that exosomes tend not to fuse indiscriminately into randomly encountered cells due to the designed membrane binding proteins and lipids that are packaged by the parent cells.^11^ Thus, M2‐Exo was further evaluated for its intracellular delivery efficiency in Mφs (Figure 2F,G). M1 Mφs were incubated with fluorescence dye‐labeled exosomes at a concentration of 0 to 100 µg mL^−1^ for 1 or 4 h. The confocal microscopy images showed a significant increase in cellular attachment and internalization of M2‐Exo in M1 Mφs within 4 h, which is acceptable to apply for Mφ‐derived exosome‐guided phenotypic switch in Mφs. M2‐Exo induced intracellular delivery to M1 Mφs in dose and time dependent manners. This result indicates that M2‐Exo is recognizable to M1 Mφs by their similarity to the donor cells in the same cell lineage. In the previous study, it was reported that the exosomes derived from mononuclear phagocytes such as monocytes, Mφs, and dendritic cells are preferentially taken up by Mφs.^12^ We also confirmed that M2‐Exo was preferentially taken up by phagocytic cells such as Mφs and dendritic cells compared to other types of cells (Figure S2, Supporting Information). Notably, M2‐Exo exhibited the highest cellular uptake efficiency in the classically activated M1 Mφs, suggesting target cell‐selective exosome‐guided cell reprogramming.

### 2.2 Exosome‐Guided Reprogramming of M1 to M2 Mϕs In Vitro

**Figure 3:**
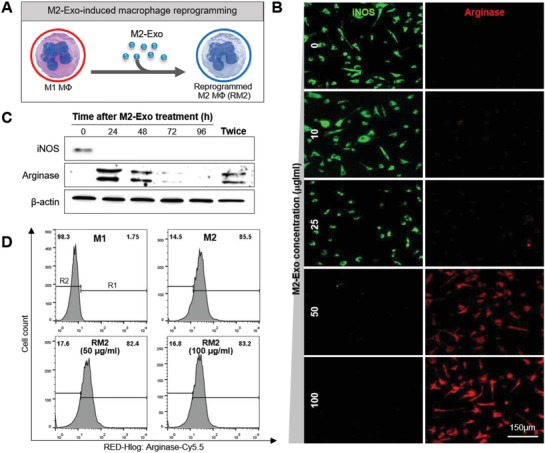
M2‐Exo‐guided direct reprogramming of M1 Mϕs to M2 Mϕs. A) Illustration of M2‐Exo‐induced Mϕ reprogramming. B) Immunostaining of iNOS and Arginase in M1 Mϕs after 24 h incubation with 10, 25, 50, and 100 µg mL^−1^ of M2‐Exo, respectively. C) Western blot analysis of M1 Mϕs treated with 50 µg mL^−1^ of M2‐Exo over time. D) FACS histogram showing reprogramming efficiency of M1 Mϕs treated with 50 and 100 µg mL^−1^ of M2‐Exo.

For regenerative medicine therapies, a number of studies have been recently reported about the therapeutic potential of exosomes derived from different types of stem cells by simply adding them to damaged tissues and organs, without an understanding of the mechanisms and characteristics of structural and functional recovery.^13^ To the best of our knowledge, this study is the first report exploring the capability of exosomes to manipulate cellular phenotypes via transferring their functionally active cargos. In order to verify whether exosomes trigger Mφ reprogramming from a proinflammatory M1 phenotype toward an anti‐inflammatory M2 phenotype, M2‐Exo was incubated with classically activated M1 Mφs in serum‐free media containing different concentrations of exosomes for 24 h (**Figure** **3**A). Immunocytochemistry (ICC) analysis showed that M1 Mφs preserved iNOS expression to some extent as M2‐Exo concentration increased (Figure 3B). At an exosome concentration of around 50 µg mL^−1^, the iNOS immunoreactive signal completely disappeared from the cells while Arginase expression was rapidly and robustly induced. This result suggested that classically activated M1 Mφs were successfully reprogrammed into M2 Mφs via M2‐Exo‐guided phenotypic switch, and the adequate threshold concentrations of exosomes (above 50 µg mL^−1^) were required to reprogram Mφs. The protein expression levels of Arginase were gradually increased with increasing concentrations of M2‐Exo over 50 µg mL^−1^ and then the Arginase production became saturated steadily. However, the survival rate of Mφs was lowered at high exosome concentration of 200 µg mL^−1^ or more (Figure S3, Supporting Information), suggesting that excessive stimulation by exosomes may cause massive negative changes in cells and thus disrupt endoplasmic reticulum (ER) homeostasis. Although ER stress is generally known to be linked to the formation and release of extracellular vesicles including exosomes,^14^ disturbance of ER homeostasis is also found to be induced by extracellular vesicles themselves.^15^

As previously mentioned, the monocyte‐Mφ lineage is known to show a remarkable degree of phenotypic plasticity referring to the capacity of activated monocytes to alter their function.^16^ Even considering the plasticity and flexibility of Mφs, it is necessary to preserve an anti‐inflammatory Mφ activation state to some degree for successful Mφ reprogramming‐based wound healing. Thus, the sustainability of M2‐Exo‐guided phenotypic switch was further investigated by western blot analysis (Figure 3C). After treating classically activated M1 Mφs with 50 µg mL^−1^ of M2‐Exo, the protein expression levels of both iNOS and Arginase were examined at various time points. As expected, within 24 h after treatment, it was clearly shown that M1‐ and M2‐specific markers were turned off and turned on, respectively. Although the iNOS expression was completely absent in RM2 Mφs after a single M2‐Exo treatment, the Arginase expression persisted for only 2 d, showing its unsettled Mφ phenotypic switch. When RM2 Mφs were additionally stimulated 48 h after the first M2‐Exo treatment, the Arginase expression lasted longer than 4 d. In consideration of the period of time for wound healing phases, it is expected that this prolonged state of Mφ reprogramming would satisfy the demand for shortening the inflammation phase. In normal skin wound healing, the inflammation stage usually lasts for 2–5 d after injury, during which the proinflammatory M1 Mφs reach their peak population in 1–2 d.^17^ Since many chronic wounds are the result of chronic inflammation lasting longer than a week,^18^ a complete and rapid conversion of excessive proinflammatory M1 to anti‐inflammatory M2 Mφs at an early stage may be effective in preventing wounds from becoming chronic. To accurately quantitate the degree of M2‐Exo‐guided Mφ reprogramming ability, a comparison of Arginase levels in classically activated M1 Mφs, alternatively activated M2 Mφs and RM2 Mφs was conducted using flow cytometry (Figure 3D). The cells turned on and off Arginase were gated to the arbitrarily selected region 1 and 2 (R1 and R2), respectively. The flow cytometry analysis showed that RM2 Mφs had almost the same level of Arginase as alternatively activated M2 Mφs. Specifically, 98.3% of classically activated M1 Mφs were observed in R2, and 85.5% and 82.4% of alternatively activated M2 Mφs and RM2 Mφs, respectively, were detected in R1. There was no significant difference in Arginase levels between 50 and 100 µg mL^−1^ M2‐Exo groups. This result suggested that almost all classically activated M1 Mφs were reprogrammed into M2 phenotype with a conversion rate of ≈96%. C57BL/6 and BALB/c mice are well known as the two main mouse strains with significantly different differentiation of Mφs.[qv: 6b] Interestingly, the reprogramming ability of exosomes was also demonstrated in C57BL/6 mice (Figures S4 and S5, Supporting Information), supporting that this exosome‐guided Mφ reprogramming method can be broadly accepted across different strains having different immune differentiation processes. Moreover, we confirmed that it could be repolarized from M2 Mφs to M1 Mφs via M1‐Exo (Figure S6, Supporting Information). This result demonstartes that Mφ‐derived exosomes can provide guidance on Mφ activation switching.

### 2.3 Identification of Major Molecules and Mechanisms Associated with M2‐Exo‐Guided Mϕ Reprogramming

**Figure 4:**
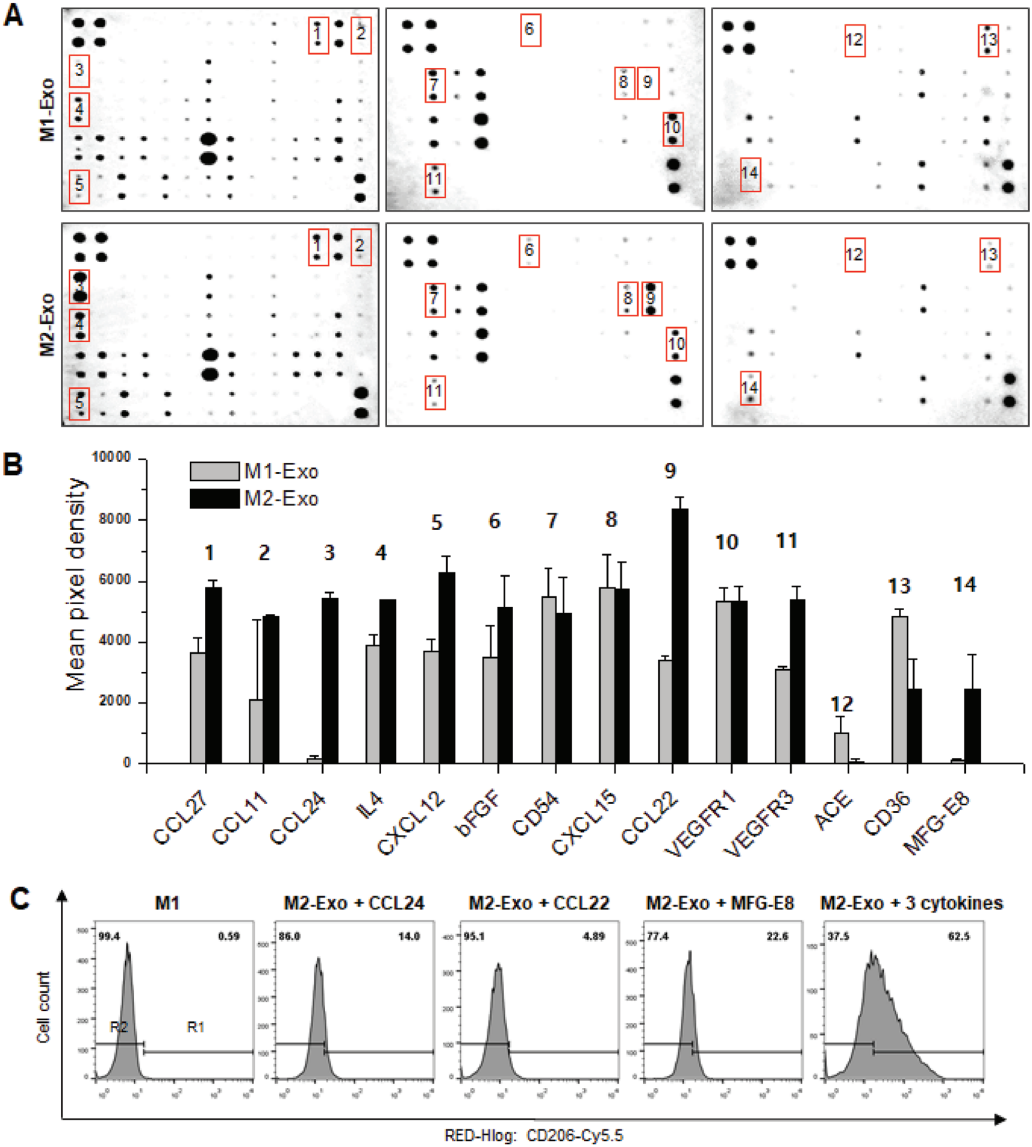
Cytokine expression profile of exosomes determined by antibody array and identification of major molecules of macrophage reprogramming. A) The representative images of cytokine antibody array. B) Comparison of mean pixel density measurement between cytokines of M1‐Exo and M2‐Exo. C) FACS histogram showing reprogramming efficiency of M1 Mϕs treated with M2‐Exo (25 µg mL^−1^) and each cytokine (100 ng mL^−1^).

Exosome is a cell‐derived vesicle known as a potential intercellular communicator that exchanges cellular substances and information. Exosomes contain a variety of different signal molecules representing their parental cells. To study key reprogramming factors, among various exosomal composition including proteins, nucleic acids, lipids, and metabolites, herein we focused more on proteins that is the most abundant exosomal cargo.^19^ Some common proteins are shared between exosomes from multiple cell types, while other specific proteins are selectively loaded into exosomes. Among different types of exosomal components, herein cytokines were evaluated to understand the main mechanism of M2‐Exo‐guided Mφ reprogramming. One hundred and forty four cytokines in both M1‐Exo and M2‐Exo were analyzed using a mouse cytokine array panel (**Figure** **4**A). First, it was confirmed that there was no difference in the IL4 expression level between M1‐exo and M2‐exo, supporting that the M2‐exo samples were not contaminated during the exosome isolation process by the proteins used in Mφs differentiation (Figure 4B). Also, the exosomal levels of the following 14 cytokines were found to be significantly different between M2‐Exo and M1‐Exo: C‐C motif chemokine 27 (CCL27), C‐C motif chemokine ligand 11 (CCL11), C‐C motif chemokine ligand 24 (CCL24), IL4, C‐X‐C motif chemokine ligand 12 also known as stromal cell‐derived factor‐1 (CXCL12, SDF1), basic fibroblast growth factor (bFGF), cluster of differentiation 54 (CD54), C‐X‐C motif chemokine ligand 15 (CXCL15), C‐C motif chemokine ligand 22 (CCL22), vascular endothelial growth factor receptor 1 (VEGFR1), vascular endothelial growth factor receptor 3 (VEGFR3), angiotensin‐converting enzyme, cluster of differentiation 36 (CD36), and milk fat globule‐epidermal growth factor (EGF) factor 8 protein (MFG‐E8). As shown in Figure 4B, various cytokines involved in wound healing were identified as being loaded at significantly higher levels in M2‐Exo compared to M1‐Exo: CCL27, CCL11, CCL24, IL4, CXCL12, bFGF, CCL22, and MFG‐E8. CCL27 accelerates skin regeneration by accumulating bone marrow‐derived keratinocytes^20^ and CCL11 promotes angiogenesis by activating the PI3K/Akt pathway.^21^, ^22^ Interestingly, CCL24, which is classified as a chemokine marker of M2 Mφs together with CCL22,^23^ was noticeably higher in M2‐Exo than M1‐Exo. It is known that Mφs can be activated and adopt distinct phenotypes by stimulating them with different cytokines. For instance, the stimulation of Mφs with Th2 cytokines such as IL10 and IL4 upregulates major chemokine ligands, such as CCL17, CCL22, and CCL24, that promote M2 Mφ differentiation.^24^ This suggests that both CCL22 and CCL24 may be key molecules directly involved in the M2‐Exo‐guided direct cell reprogramming. IL4, CXCL12, and bFGF are also well‐known major cytokines and growth factors participating in the wound healing process.^25^ Notably, MFG‐E8, that are capable of promoting the switch from M1 to M2 Mφs,^26^ was detected at a 21‐fold higher level in M2‐Exo compared to M1‐Exo. In addition, it was reported that MFG‐E8‐stimulated Mφs accelerate wound healing by promoting the expression of bFGF and attenuating the proinflammatory.^27^ The expression levels and the reprogramming ability of three major reprogramming cytokines (CCL24, CCL22, and MFG‐E8) were further investigated by western blot (Figure S7, Supporting Information) and FACS analysis (Figure 4C). To further identify the key player among the three reprogramming factors, M1 Mφs incubated with 25 µg mL^−1^ of M2‐Exo, which is lower than the critical concentration (50 µg mL^−1^), were treated with CCL24, CCL22, and MFG‐E8, respectively. As shown in Figure 4C, exosome‐guided direct reprogramming was induced by various cytokine cocktails rather than by a single protein cytokine, which is presumed to be a major reprogramming factor expressed highly in M2‐Exo. Taken together, these results demonstrate that M2‐Exo carry key reprogramming factors with various wound healing factors, offering additional benefits for M2‐Exo‐guided Mφ reprogramming in wound healing applications.

### 2.4 Enhanced Fibroblast Migration and Endothelial Cell Tube Formation by the Reprogrammed M2 Mϕs

**Figure 5:**
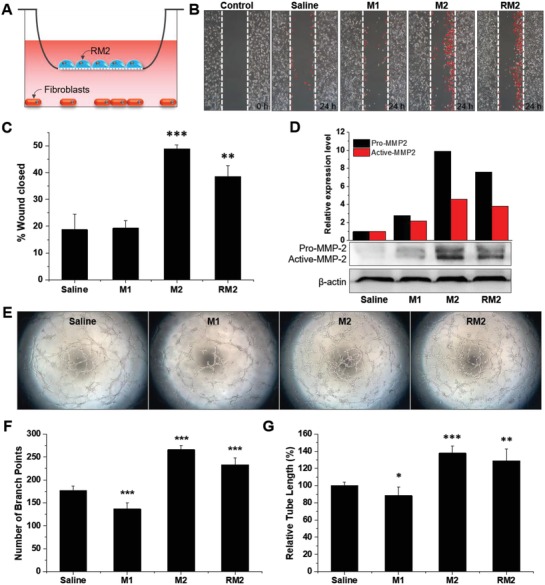
In vitro therapeutic effects of RM2 Mϕs. A) Illustration of cocultures of fibroblasts and Mϕs. B) Representative phase‐contrast images of wounded fibroblasts cocultured with Mϕs. C) Quantification of wound closure among the three types of Mϕ groups. *n* = 3; **p < 0.01, and ***p < 0.001 versus saline. D) Levels of MMP‐2 in Mϕ/fibroblast coculture supernatant at 24 h after inflicting the wounds. E) Representative pictures of tube formation assay of vascular endothelial cells cocultured with Mϕs. F,G) Quantitative evaluation of total number of branches and tube length at 24 h after coculturing endothelial cells and Mϕs. *n* = 5; *p < 0.05, **p < 0.01, and ***p < 0.001 versus saline.

In the second wound healing stage, called proliferation phase, alternatively activated M2 Mφs are mainly involved in proliferation and migration of fibroblasts and angiogenesis to terminate inflammation and induce wound repair.^28^ Thus, herein we preferentially evaluated in vitro wound healing effects of RM2 on the wound healing activity of fibroblasts and on the angiogenic capability of endothelial cells (**Figure** **5**). First the pattern of fibroblast migration was examined by performing in vitro scratch wound healing assay. After inflicting scratch wounds across confluent fibroblast monolayers, fibroblasts were cocultured with classically activated M1 Mφs, alternatively activated M2 Mφs or RM2 Mφs (Figure 5A). As expected, the fibroblasts cocultured with alternatively activated M2 Mφs showed the fastest cell migration, resulting in about 2.5‐fold faster wound healing rate compared to the saline group. In contrast, the M1 group was not significantly different from the saline group (Figure 5B,C). The RM2 group also showed a fast wound closure rate similar to that of the M2 group. It has been known that fibroblasts produce and secrete proprotective factors such as MMP‐2 to promote their migration to the wound site through the basement membrane during extracellular matrix (ECM) remodeling.^29^ To confirm the correlation between enhancement of wound closure by RM2 Mφs and production of MMP‐2, the protein expression levels of MMP‐2 were evaluated in a medium in which Mφs and fibroblasts were cocultured (Figure 5D). Similar to the wound scratch assay results, both alternatively activated M2 Mφs and RM2 Mφs exhibited significantly elevated expression levels of MMP‐2, compared to classically activated M1 Mφs or saline. Taken together, these results demonstrate that RM2 Mφs can contribute to enhancing the migration ability of fibroblasts by relying on MMP‐2‐mediated proteolysis, which is comparable to M2 Mφs.

Next, to assess the role of RM2 Mφs in angiogenesis, in vitro tube formation assays were performed by coculturing mouse vascular endothelial cells with classically activated M1 Mφs, alternatively activated M2 Mφs or RM2 Mφs on a Matrigel matrix (Figure 5E–G). Compared with the saline group, a marked increase in endothelial tube formation was observed in both M2 and RM2 groups. In the total number of branch points and the relative tube length, alternatively activated M2 Mφs or RM2 Mφs exhibited ≈150% or 131% and 138% or 129% increases relatively to saline, respectively. However, classically activated M1 Mφs caused a rather reduced tube formation; their total number of branch points and relative tube length decreased by 77% and 88%, respectively, compared to saline. This result suggests that persistence of an unrestrained M1 Mφ population by the failure to switch from M1 to M2 phenotypes impairs the process of angiogenesis, leading to a delay in normal wound healing as well as granulation tissue maturation. VEGF is well known to play a key role in angiogenesis and to be expressed in vascular endothelial cells through both paracrine and autocrine mechanisms.^30^ Therefore, the VEGF protein expression pattern in mouse vascular endothelial cells was further examined using western blot analysis after coincubation with classically activated M1 Mφs, alternatively activated M2 Mφs or RM2 Mφs (Figure S8, Supporting Information). The VEGF expression in vascular endothelial cells was increased in both M2 and RM2 groups and decreased in the M1 group, compared with the normal saline group. This M2 Mφ‐mediated upregulation of VEGF in vascular endothelial cells can be attributed to its overloaded exosomal cytokine bFGF (Figure 4), that mainly modulates endothelial cell expression of VEGF through paracrine mechanism of action. It is also known that in vivo the endothelial cells of mature vessels downregulate VEGF, but bFGF‐2 stimulation promotes VEGF expression in those of newly forming capillaries.^31^ In general, alternatively activated M2 Mφs are typically colocalized with endothelial branch points to promote endothelial tube formation.^32^ The confocal image of ICC clearly showed that RM2 cocultured with vascular endothelial cells was localized mostly at branching points and became part of the tubular network (Figure S9, Supporting Information), which may lead to a close crosstalk between two cells.

### 2.5 Wound Healing Effects of In Situ Exosome‐Guided Phenotypic Switch to M2 Mϕs

**Figure 6:**
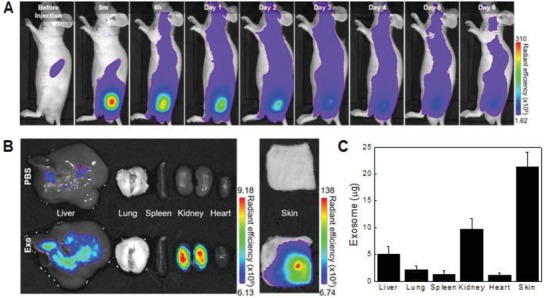
In vivo biodistribution of exosomes. A) Real‐time in vivo imaging of Cy5.5‐NHS labeled exosome. The mice were analyzed at the indicated times after subcutaneous injection of phosphate‐buffered saline (PBS) and 100 µg per 100 µL of exosomes. B) Ex vivo imaging of skins and major organs at day 2 after mice had been treated with exosomes. C) Tissue distribution of exosomes at day 2 after subcutaneous injection of exosomes.

**Figure 7:**
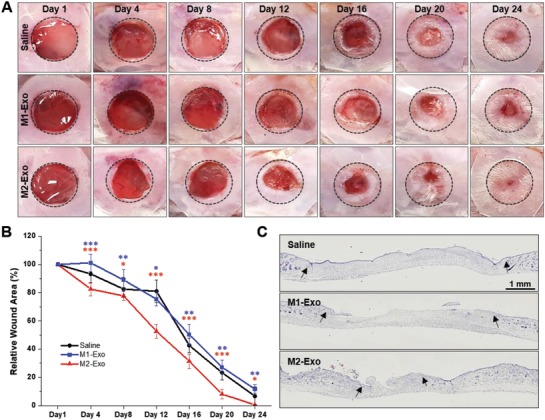
M2 Mϕs‐derived exosomes promoted wound healing in vivo. A) Representative images of wound closure after local injection of saline, M1‐Exo, and M2‐Exo. B) Wound area at every 4 d postwounding. *n* = 4; *p < 0.05, **p < 0.01, and ***p < 0.001 versus saline. C) Representative photoimages of whole wound sections injected with saline, M1‐Exo, and M2‐Exo at day 24 postwounding. The black arrows indicate the wound margins.

Prior to exploring wound healing effects of exosomes, in vivo biodistribution of exosomes was investigated by real‐time fluorescence imaging analysis (**Figure** **6**). The fluorescence signal of Cy5.5‐N‐hydroxysuccinimide (NHS) labeled exosomes remained well in the subcutaneous tissue for more than 2 d and was reduced gradually (Figure 6A). On the day 4 after subcutaneous injection, the signal was completely diminished to less than 10%. On the second day after subcutaneous injection of exosomes, the tissue distribution of exosomes showed that the largest amount of exosomes still accumulated in the skin (Figure 6B,C). This result supports that the subcutaneously injected M2‐Exo can provide sufficient time to induce local exosome‐guided macrophage reprograming. Exosomes had accumulated in the kidneys but had not reached the concentrations identified in the previous section. To drive wound‐resident proinflammatory M1 Mφs on‐site toward anti‐inflammatory M2 phenotype, M2‐Exo was applied directly to an excisional skin wound in mice via subcutaneous injection around the wound; Each exosome derived from classically activated M1 Mφs or alternatively activated M2 Mφs was treated twice to wound site day 1 and day 4 post full‐thickness skin excision (**Figure** **7**). First, in vivo wound repair ability of M2‐Exo was examined by visual assessment of excisional wound closure and wound area measurement (Figure 7A,B). When compared to the saline‐treated control group, the M2‐Exo‐treated group showed significantly accelerated wound closure even already on day 4. This successful primary wound closure may be mainly attributed to the rapid switch of proinflammatory M1 Mφs to anti‐inflammatory M2 Mφs via M2‐Exo‐guided Mφ reprogramming. In general, early wound closure is a major determinant of wound outcomes because many conventional wound treatments such as cytokines and growth factors provide only a marginal benefit to wound healing due to a delayed primary wound closure.^33^ In this regard, the treatment of M1‐Exo showing delayed primary wound closure somewhat deteriorated overall wound healing likely due to the prolonged inflammation stage by reinforcing M1 Mφ phenotype. As a result, the wounds treated with M2‐Exo displayed pronounced wound healing efficacy throughout the entire wound healing process, achieving complete wound closure by day 24. The microscopic examination of hematoxylin and eosin (H&E)‐stained wound sections also revealed that the M2‐Exo‐treated wounds had more granulation tissue and thicker dermal layers with the flat epidermal surface, compared to saline or M1‐Exo‐treated groups (Figure 7C).

**Figure 8:**
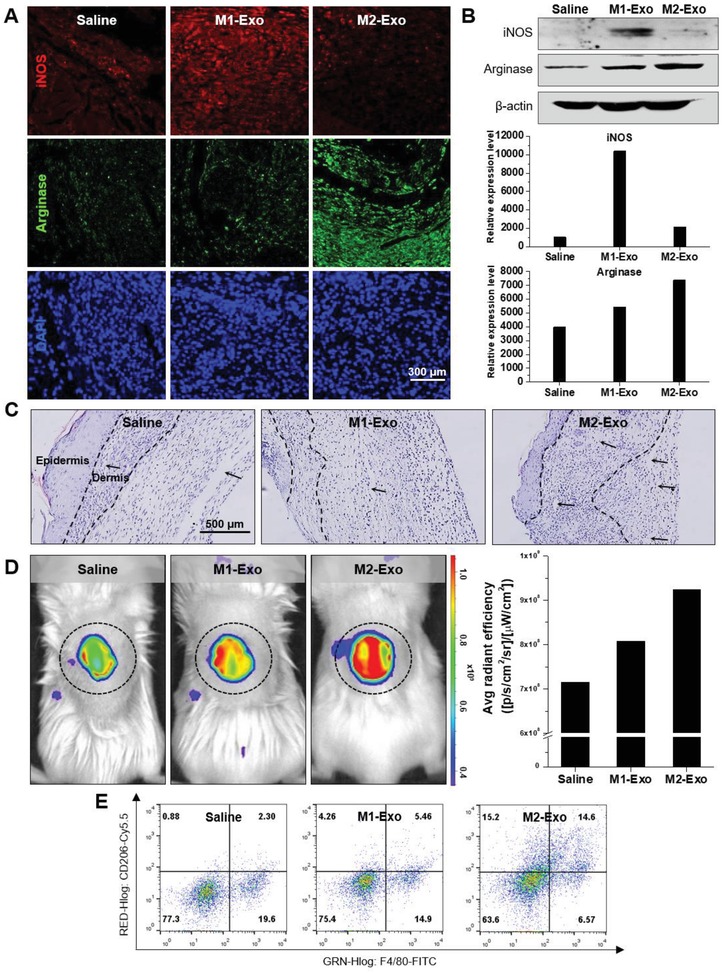
In vivo therapeutic effects of RM2 Mϕs. A) Representative immunostaining images of M1 (iNOS) and M2 (Arginase) Mϕs in mice skin tissue at 8 d postwounding. B) Western blot analysis of the expression of iNOS and Arginase in mouse skin tissues with PBS, M1‐Exo, and M2‐Exo (100 µg per 100 µL). C) Representative magnified photographs of wounds (H&E staining) 24 d after injury and subcutaneous injection of PBS, M1‐Exo, and M2‐Exo. The dotted line indicates the dermal region and the black arrow represents collagen fibers. D) Fluorescence image of MMP‐2 activity surrounding the wound bed using MMP‐2‐activatable probe. E) Representative flow plots of M1 and M2 Mϕs population in the wound 5 d after injury and subcutaneous injection of PBS, M1‐Exo, and M2‐Exo.

To explore in vivo M2‐Exo‐guided reprogramming of proinflammatory M1 Mφs into anti‐inflammatory M2 Mφs, immunohistochemical studies were additionally performed using antibodies specific for M1 and M2 Mφs markers (iNOS and Arginase) (**Figure** **8**A). With M2‐Exo treatment, the expression of iNOS and Arginase was markedly reduced and enhanced, respectively. Comparably, a significant increase and decrease in iNOS and Arginase expression, respectively, were observed in M1‐Exo‐treated wounds. The altered Mφ‐specific marker expression pattern was also confirmed by western blotting on the eighth day after the first treatment of exosomes (Figure 8B). These results reveal that local proinflammatory M1 Mφs were directly convert into anti‐inflammatory M2 Mφs with M2‐Exo treatment. As shown in Figure 8C, wound dermal cellularity and collagen production were significantly increased in the M2‐Exo‐treated group compared to the saline group, supporting that the successful local switch of M1 Mφs to M2 Mφs accelerated re‐epithelialization. MMP has a fundamental role in ECM remodeling and re‐epithelialization by promoting migration of various cell types such as skin fibroblast and keratinocytes.^34^ To observe the MMP‐2 activity at the wound site in real time, a bioactivatable probe specific for MMP‐2 activity was applied to the wounds 4 d after exosome treatment. The saline and M1‐Exo group exhibited a slight fluorescence signals at the wound margin, whereas the M2‐Exo‐treated group showed dramatically enhanced MMP‐2 activity (Figure 8D). Finally, to explore population changes of M1 and M2 Mφs in the wound region, flow cytometry analysis was performed (Figure 8E). Although it was not as efficient as in vitro tests, major changes in local M1 and M2 Mφ population in the M2‐Exo treated group was observed, supporting that M1 Mφs were successfully reprogrammed into M2 Mφs in vivo. Taken together, these results demonstrated that the effective on‐site switch to M2 Mφ polarization via M2‐Exo‐guided cell reprogramming could exert a favorable outcome in skin wound repair by improving the speed and quality of wound healing.

## 3 Conclusion

To the best of our knowledge, this study is the first report that exosomes derived from alternatively activated M2 Mφs can promote cutaneous wound repair by inducing in situ direct conversion of classically activated M1 Mφs into reprogrammed M2‐like phenotype. The role of M2‐Exo is not only to act on phenotypic switch of Mφs but also to influence the surrounding cells through the paracrine secretion to create the desired environment. M2‐Exo possesses crucial regulators (CCL22, CCL24, and MFG‐E8) of switching M1 Mφs toward M2 Mφs as dominant luminal components. Other abundant luminal‐cytokines and ‐growth factors such as IL4, CXCL12, and bFGF can also contribute to improved wound healing and repair by enhancing angiogenesis and re‐epithelialization. As a result, M2‐Exo‐guided Mφ reprogramming could rapidly and very efficiently reinforce the M2 Mφ phenotype at the wound site with an accompanying attenuation of M1 Mφ population, providing remarkable advantages particularly in primary wound closure. Since exosome‐guided phenotypic switch of Mφs is a bilateral strategy between M1 and M2 Mφs, it offers a promising therapeutic strategy for the treatment of various diseases associated with an imbalance between proinflammatory and anti‐inflammatory immune responses.

## 4 Experimental Section

*Cell Lines*: Bone marrow derived‐macrophages (BMDM) from Balb/c and C57BL/6 mice was prepared as previously described with minor modifications.^35^ Briefly, bone marrow was isolated from femur and tibia bones using 21G needle and 10 mL syringe and cultured for 7 d in Roswell Park Memorial Institute 1640 Media (Gibco) with 10% fetal bovine serum (Gibco), penicillin/streptomycin (100 µg mL^−1^) (Gibco), and 10 ng mL^−1^ M‐CSF. For M1 activation, cells were maintained for 2 d with 40 ng mL^−1^ IFN‐γ (Peprotech) and for M2 activation, cells were treated for 2 d with 20 ng mL^−1^ IL‐4 (Peprotech). For wound scratch migration assay, NIH‐3T3 fibroblasts (ATCC 30‐2003 [American Type Culture Collection, Manassas, VA, USA]) were used. SVEC4‐10 endothelial cells (ATCC CRL‐2181 [American Type Culture Collection, Manassas, VA, USA]) were used to perform tube formation assay.

*Isolation and Characterization of Exosomes Derived from BMDM*: Exosomes were isolated using ultracentrifugation method as previously described.^36^ Briefly, bone marrow‐derived macrophages were incubated for 2 d in serum‐free conditions. After incubation for 48 h, the cell culture supernatant was collected and centrifuged at 300 g for 10 min at 4 °C to remove cells and then centrifuged at 2000 g for 10 min to remove of dead cells. After centrifugation at 10 000 × *g* for 30 min to remove cell debris, the supernatant was harvested and centrifuged at 150 000 × *g* for 90 min to collect the exosome pellet. The pellet was washed with phosphate‐buffered saline (PBS) to remove contaminating proteins and centrifuged again at 150 000 × *g* for 90 min. Measurements of exosome size was performed with dynamic light scattering (DLS, Zetasizer Nano, Malvern Instruments, UK). For TEM analysis of exosomes, exosomes were fixed in 2% paraformaldehyde solution overnight. Exosome solution was centrifuged for 30 min at 150 000 × *g* and then suspended in absolute ethanol. 2 µL of exosome suspension was transferred onto Formvar‐carbon coated electron microcopy grids. The grid was contrasted with uranium acetate solution for 1 min and then examined using TEM (Tecnai F20 G^2^).

*Exosome Labeling and Cellular Uptake*: The exosomes derived from macrophages(Mφs) were labeled with a membrane labeling dye (1′‐dioctadecyl‐3,3,3',3'‐tetramethylindodicarbocyanine, DiD) (ThermoFisher scientific) according to the manufacturer's protocol. The exosomes were incubated in a 5 µg mL^−1^ DiD staining solution at 37 °C for 30 min. 300 kDa ultrafiltration tubes (Sigma‐Aldrich) were used to remove unattached dyes and the labeled exosomes were ultracentrifuged at 100 000 × *g* for 70 min, washed with PBS, and ultracentrifuged again. Mφs were seeded at density of 3 × 10^5^ cells in 35 mm confocal dish, incubated at 37 °C with labeled exosomes (10, 25, 50, and 100 µg mL^−1^) for 1 h, and then observed with a confocal microscope (Leica TCS SP6, Germany). The fluorescence intensities were quantified using an image analyzer.

*Immunocytochemistry and Flow Cytometry*: Cells were rinsed with PBS and fixed with 4% paraformaldehyde for 10 min. After fixation, cells were washed three times with ice cold PBS and permeabilized with 0.25% Triton X‐100 (PBST) for 5 min. Blocking with PBST solution containing 1% bovine serum albumin (BSA) was performed for 30 min and primary antibodies were added in blocking solution for overnight at 4 °C. The cells were washed three times with PBST and then the secondary antibodies were incubated in the dark for 1 h at room temperature. After three PBST washes, 4′,6‐diamidino‐2‐phenylindole (DAPI) staining was performed. The images were taken TCS SP5 confocal microscope (LEICA, Germany). For flow cytometry analysis, the macrophages were fixed with 4% paraformaldehyde and permeabilized with 0.25% Triton X‐100 (PBST). Then, primary antibodies were added in 5% BSA and incubated for 2 h at 4 °C. After two PBST washes, cells were stained with secondary antibodies in the dark for 1 h at 4 °C. The stained macrophages were analyzed by flow cytometry (Guava easyCyte Flow Cytometers, MERCK). The flow cytometry for in vivo study was performed using the CD11b MicroBeads kit (MACS, # 130‐049‐601) according to the manufacturer's instructions. The antibodies used in this study are as follows: iNOS (abcam, ab210823, 1:200), Arginase (CST, #93668, 1:200), CD206 (Santa Cruz Biotechnology, SC‐376108, 1:200), F4/80 (abcam, ab105155, 1:200) Alexa Fluor 488 (abcam, ab150117, 1:500), Alexa Fluor 647 (abcam, ab150083, 1:500).

*Western Blot Analysis*: Cell lysates and mouse skin homogenates were quantitated by bicinchoninic acid protein assay and electrophoresed with 8–12% sodium dodecyl sulfate polyacrylamide gel electrophoresis gel and transferred to polyvinylidene difluoride membrane (Millipore). Proteins transferred to the membrane were blocked with 5% skim milk for 30 min at room temperature and subsequently incubated with primary antibodies overnight at 4 °C. The membranes were washed three times for 15 min with TBST (Tris‐buffered saline, 0.1% Tween 20) and incubated for 1 h at room temperature in a blocking solution with horseradish peroxidase tagged second antibodies the labeled proteins were visualized with LAS‐3000 Luminescent Image Analyzer (FujiFilm, Tokyo, Japan). The antibodies used in this study are as follows: CD86 (abcam, ab112490, 1:200), iNOS (abcam, ab210823, 1:200), Arginase (CST, #93668, 1:200), CD206 (Santa Cruz Biotechnology, SC‐376108, 1:200), β‐actin (abcam, ab8227, 1:1000), CD63 (Santa Cruz Biotechnology, SC‐15363, 1:200), Alix (abcam, ab117600, 1:200), MMP2 (CST, #4022, 1:200), and VEGF (Santa Cruz Biotechnology, SC‐7269, 1:200).

*Cytokine Antibody Array*: Cytokine expression profiles in the exosome derived from macrophage were detected using mouse Cytokine Antibody Array C2000 (RayBiotech) and semiquantified following the manufacturer's instructions. The immunoblot images were visualized and captured using LAS‐3000 Luminescent Image Analyzer (FujiFilm, Tokyo, Japan) and the intensity of each spot in membranes was analyzed using ImageJ software (NIH).

*Wound Scratch Migration Assay*: NIH‐3T3 cells were plated in six well culture plate (SPL Life Sciences, Gyeonggi‐do, Korea) at a density of 2 × 10^5^ cells per well in fresh culture medium. Cells were incubated at 37 °C and 5% CO_2_ overnight to allow formation of confluent monolayer at 70% confluence. Subsequently, M1, M2, or reprogrammed M2 Mφs (RM2) were added to each well at a density of 2.5 × 10^5^ cells per well. After incubating cocultured cells for additional 12 h, cell layers were scratched with 200 µL pipette tip and carefully washed with PBS prior to taking microscopic images with CK40 culture microscope (Olympus, Tokyo, Japan) at 0 or 24 h. All experiments were carried out in quadruplicates.

*Tube Formation Assay*: In 96‐well plates, 50 µL of Matrigel (BD Biosciences) was added and allowed to solidify at 37 °C for 30 min. Subsequently, SVEC4–10 endothelial cells (ATCC CRL‐2181 (American Type Culture Collection, Manassas, VA, USA) were seeded at a density of 2 × 10^4^ cells per 100 µL. Also, each macrophages were cocultured at a density of 4 × 10^4^ cells per 100 µL. After 24 h, the formation of the tube was captured with a CK40 culture microscope (Olympus, Tokyo, Japan), and the number of branches and tube length were analyzed by ImageJ software (NIH).

*In Vivo Biodistribution Study of Exosomes*: For the in vivo biodistribution test, PBS or exosomes (100 µg per 100 µL) was subcutaneously injected into BALB/c nude mice (*n* = 3). The fluorescence intensity was measured by in vivo imaging system (IVIS) Lumina Series III (PerkinElmer, Massachusetts, USA). The mice were sacrificed at 48 h after injection, the skin and other organs including liver, lung, spleen, kidney, and heart were collected and lysed using radio‐immunoprecipitation assay buffer. The fluorescence intensities of skin and tissue lysates were measured by IVIS Lumina Series III. Quantification of fluorescence intensities in tumor region of interest (ROIs) were analyzed using Living Image software (PerkinElmer, Massachusetts, USA).

*Wound Healing Model and Treatment*: Balb/c mice (male, 5 weeks old) for skin wound model were purchased from Nara Biotech (Seoul, Korea). All animal experiments were performed in accordance with the International Guide for the Care and Use of Laboratory Animals and approved by Korea Institute of Science and Technology. After mice were anesthetized first, the back of mouse was shaved with an electric clipper and wiped 70% ethanol and dry. Using a sterile biopsy punch (8 mm size), the biopsy punch was pushed through the middle part of the back skin and twisted to the left and right to penetrate the back skin of the anesthetized mice. Wound closure photographs were taken every 4 d and 100 µg per 100 µL exosomes were treated on day 1 and day 4. The diameter of each wound was measured using Photoshop (version CS6.0; Adobe system, San Jose, CA) to calculate the percentage of wound closure.

*Immunofluorescence*: On the eighth day after the first exosome treatment, the back skins of the mice were cut off. The incised mouse skin was fixed and embedded in an optical cutting temperature compound (Fisher scientific). The embedded tissues were cut to a thickness of 10 µL using a freezing microtome (LEICA CM‐1900, Germany) and permeabilized and blocked with 2% BSA containing 0.5% Triton X‐100. The images were taken TCS SP5 confocal microscope (LEICA, Germany).

*Synthesis of MMP‐2 Probe*: The nanosensor peptide (Cy5.5‐Gly‐Pro‐Leu‐Gly‐Val‐Arg‐Gly‐Lys(BHQ3)‐GlyGly‐OH) was synthesized using solid‐phase Fmoc peptide chemistry as previously described.^37^ Briefly, The Dde protecting group was selectively removed from Fmoc‐Gly‐Pro‐Leu‐Gly‐Val for site specific binding of BHQ‐3 (Biosearch Technologies, Novato, CA) to the primary amine of the lysine adjacent to the cleavable sequence by addition of 2% hydrazinein *N*,*N*‐dimethyformamide (DMF). The peptide resin was then extensively washed after addition of BHQ‐3 succinimide ester in anhydrous DMF. The final Fmoc group was then removed and the Cy5.5 succinimide ester (Amersham Bioscience, Piscataway, NJ, USA) was conjugated to the N‐terminal glycine. After the peptide was deprotected and cleaved from the resin using trifluoroacetic acid, the resulting fluorescence peptide was purified by reverse‐phase high performance liquid chromatography.

*Visualization of In Vivo MMP2 Activity*: The wound was treated with saline, 100 µg per 100 µL M1‐Exo, M2‐Exo on the first day, and topical application of 1 mg per 30 µL MMP2 probe solution with the same treat on the fourth day. After that, the wounds were sealed with Tegaderm dressing and Cova elastic bandage. The next day, the bandage was cut out and the wound was washed with PBS to remove the remaining MMP‐2 solution and the fluorescence images were taken using the IVIS II Lumina device (Caliper Life Sciences, Hopkinton, MA).

*Statistical Analysis*: All data were expressed as mean ± SEM from at least three independent experiments. All groups were compared with student *t*‐test or one‐way analysis of variance, and *p*‐values less than 0.05 were considered statistically significant.

## Conflict of Interest

The authors declare no conflict of interest.

## Supporting information

Supplementary
