# Supplementary material for: Exosome‐Guided Phenotypic Switch of M1 to M2 Macrophages for Cutaneous Wound Healing
Source: Adv Sci (Weinh). 2019 Aug 27;6(20):1900513. doi: 10.1002/advs.201900513 (PMC6794619; doi:10.1002/advs.201900513)
Supplement: Supplementary file 1 — Supplementary [file ADVS-6-1900513-s001.pdf]

Copyright WILEY-VCH Verlag GmbH & Co. KGaA, 69469 Weinheim, Germany, 2019.

ADVANCED SCIENCE
Open Access

Supporting Information

for *Adv. Sci.,* DOI: 10.1002/advs.201900513

Exosome-Guided Phenotypic Switch of M1 to M2 Macrophages for Cutaneous Wound Healing

Hyosuk Kim, Sun Young Wang, Gijung Kwak, Yoosoo Yang, Ick Chan Kwon, and Sun Hwa Kim**

Copyright WILEY-VCH Verlag GmbH & Co. KGaA, 69469 Weinheim, Germany, 2019.

Supporting Information

Exosome-Guided Phenotypic Switch of M1 to M2 Macrophages for Cutaneous Wound Healing

Hyosuk Kim, Sun Young Wang, Gijung Kwak, Yoosoo Yang, Ick Chan Kwon, and Sun Hwa Kim**

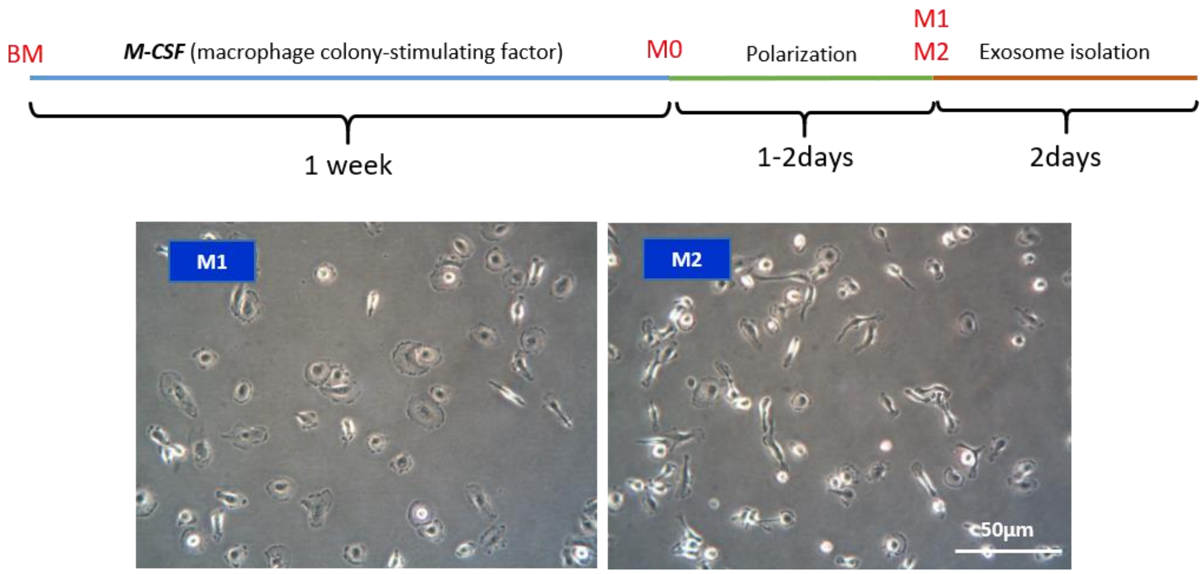

Figure S1. Establishment of M1 and M2 Mφs. Experimental schedules of Mφ polarization and the differences in morphology of M1 and M2 Mφs.

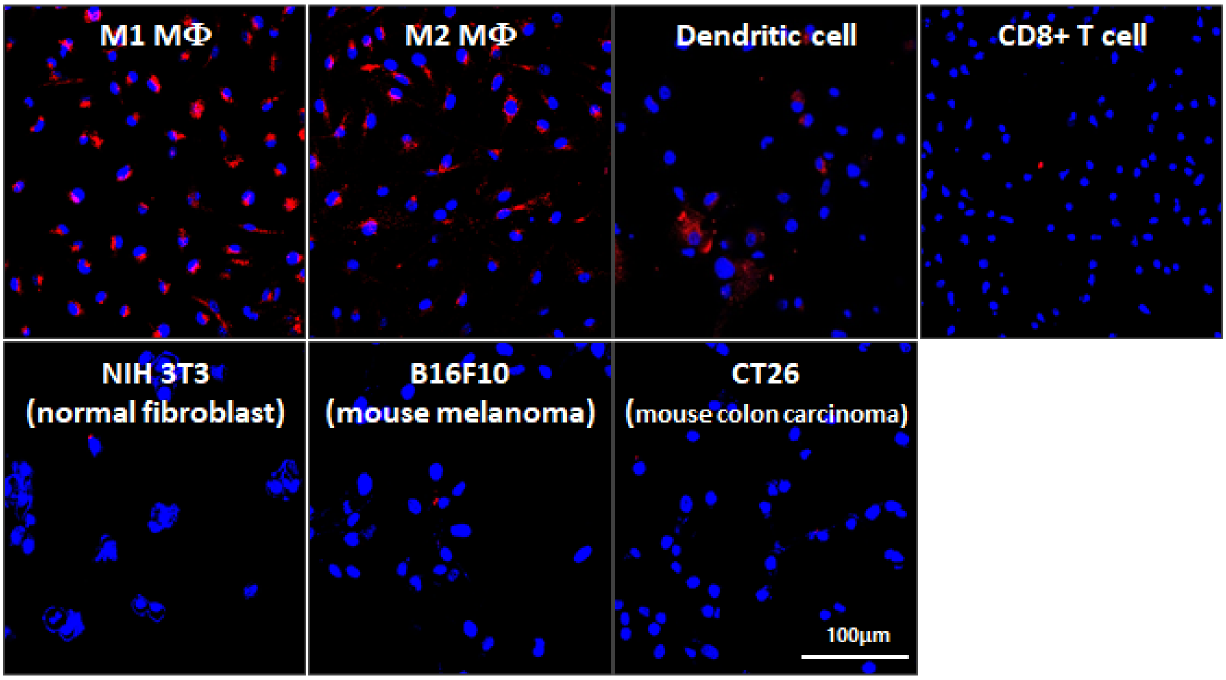

Figure S2. Confocal images of various types of cells after 4 h incubation with 50 µg/ml of DiD-labeled M2-Exo. Images of DiD-labeled M2-Exo (red) with DAPI (blue) were visualized by merging the confocal images.

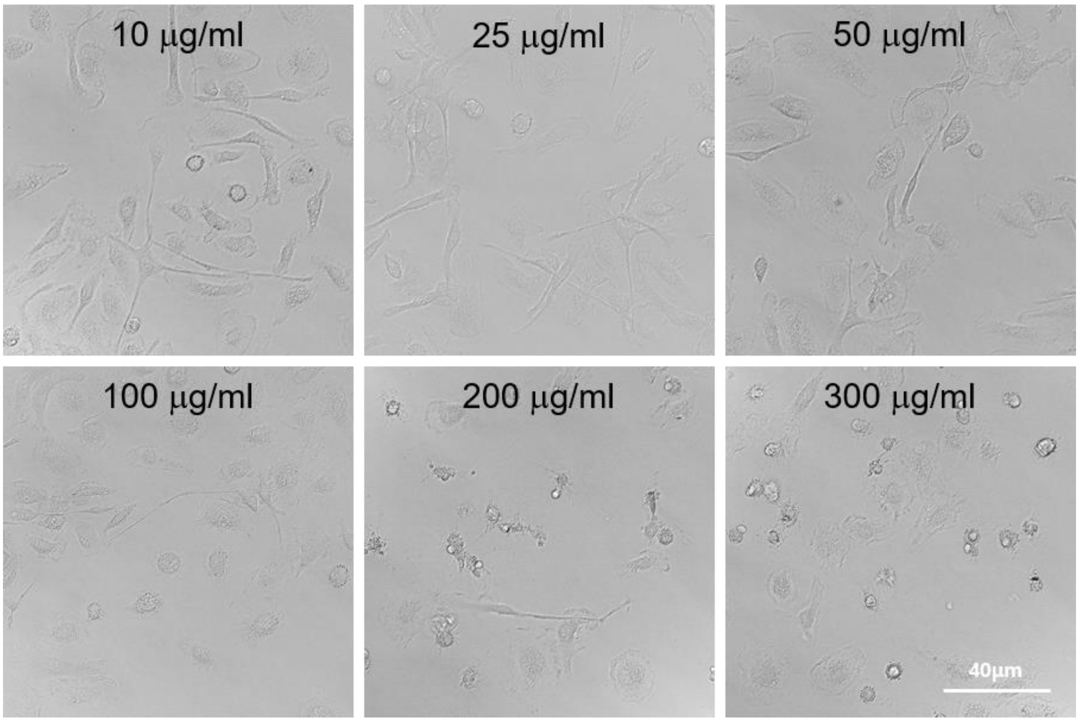

Figure S3. Morphological change of Mφs with exosome concentration. Representative bright-field micrographs of Mφs after 24 h incubation with 10, 25, 50, 100, 200 and 300 μg/ml of M2-Exo.

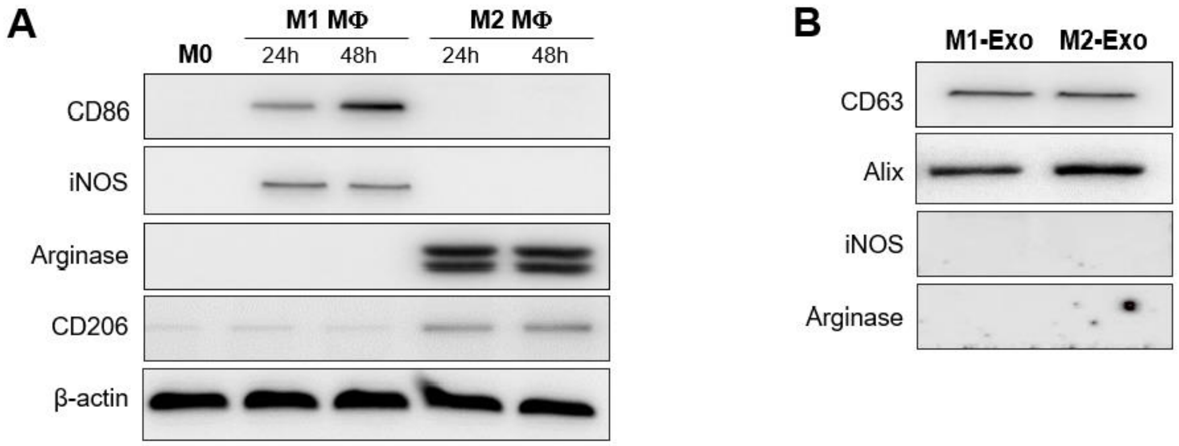

Figure S4. Establishment of M1 and M2 Mφs of C57BL/6 mice, and identification of exosomes released from Mφs. A) Western blot analysis demonstrating differences in expression of Mφ markers by polarization time. B) Western blot analysis of exosomes. Equal amounts of total proteins extracted from exosomes were immunoblotted for CD63, Alix, iNOS, and Arginase.

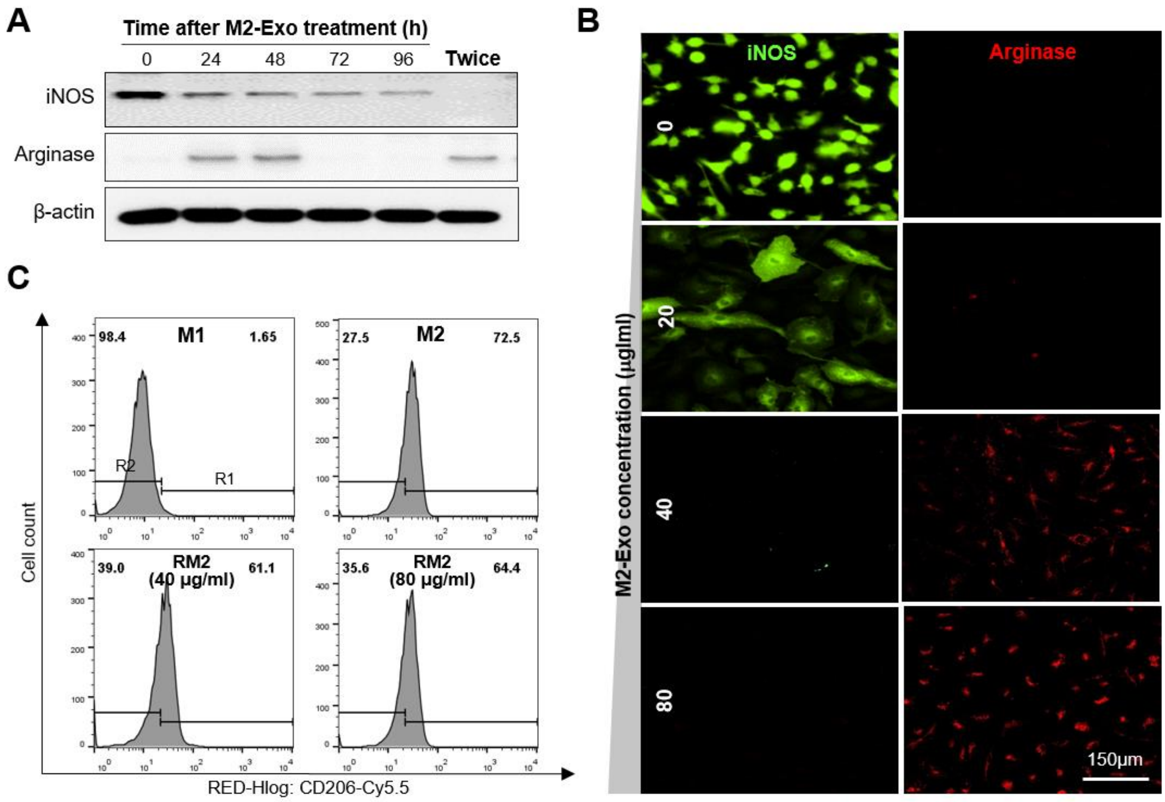

Figure S5. M2-Exo-guided direct reprogramming using Mφs from C57BL/6 mice. A) Western blot analysis of M1 Mφs treated with 40 µg/ml of M2-Exo over time. B) Immunostaining of iNOS and Arginase in M1 Mφs after 24 h incubation with 20, 40 and 80 µg/ml of M2-Exo, respectively. C) FACS histogram showing reprogramming efficiency of M1 Mφs treated with 40 and 80 µg/ml of M2-Exo.

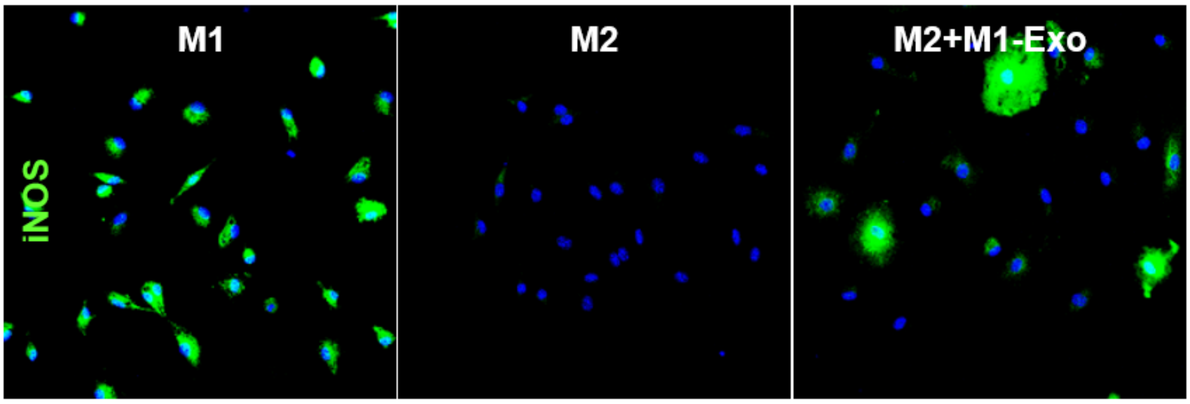

Figure S6. M1-Exo-guided direct reprogramming of M2 Mφs to M1 Mφs. Immunostaining of iNOS in M2 Mφs after 24 h incubation with 40 µg/ml of M1-Exo.

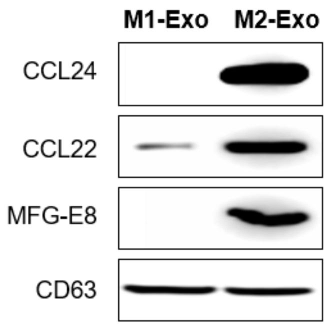

Figure S7. Western blot analysis of M1 and M2-derived exosomes. Equal amounts of total proteins extracted from exosomes were immunoblotted for CCL24, CCL22, MFG-E8 and CD63.

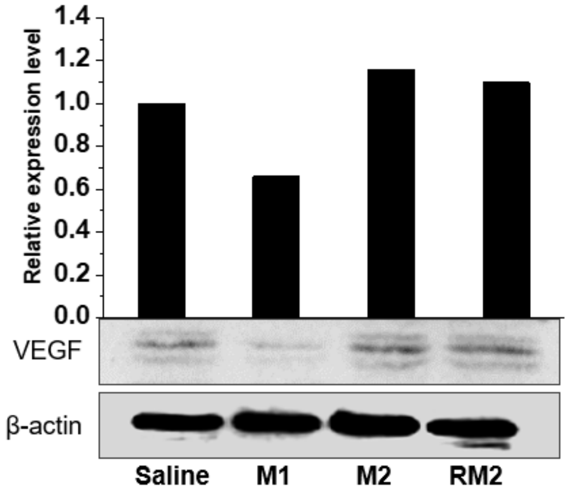

Figure S8. Western blot analysis of VEGF among the three different types of Mφ groups.

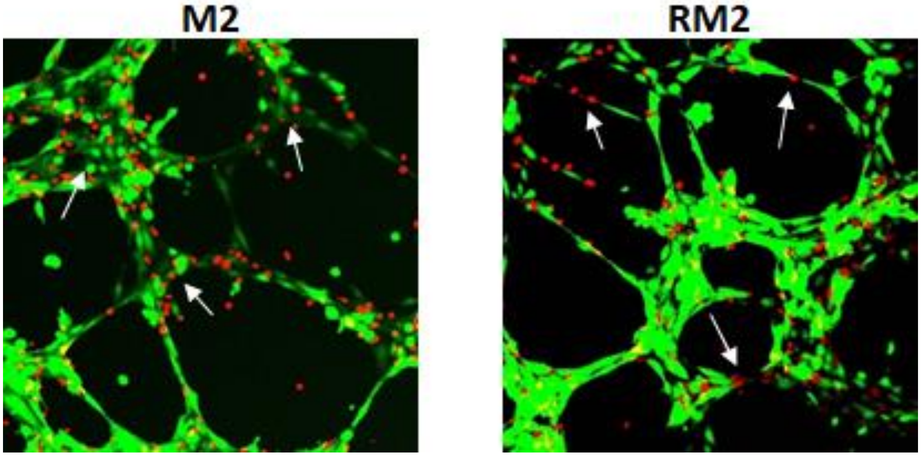

Figure S9. Co-localization of fluorescently labeled endothelial cells (green) and Mφs (red). Arrows indicate Mφs merging into the tubular structures.
